# Genome sequence of *Frateuria aurantia* type strain (Kondô 67^T^), a xanthomonade isolated from *Lilium auratium* Lindl.

**DOI:** 10.4056/sigs.4338002

**Published:** 2013-10-02

**Authors:** Iain Anderson, Huzuki Teshima, Matt Nolan, Alla Lapidus, Hope Tice, Tijana Glavina Del Rio, Jan-Fang Cheng, Cliff Han, Roxanne Tapia, Lynne A. Goodwin, Sam Pitluck, Konstantinos Liolios, Konstantinos Mavromatis, Ioanna Pagani, Natalia Ivanova, Natalia Mikhailova, Amrita Pati, Amy Chen, Krishna Palaniappan, Miriam Land, Manfred Rohde, Elke Lang, John C. Detter, Markus Göker, Tanja Woyke, James Bristow, Jonathan A. Eisen, Victor Markowitz, Philip Hugenholtz, Nikos C. Kyrpides, Hans-Peter Klenk

**Affiliations:** 1 DOE Joint Genome Institute, Walnut Creek, California, USA; 2 Los Alamos National Laboratory, Bioscience Division, Los Alamos, New Mexico, USA; 3 Theodosius Dobzhansky Center for Genome Bionformatics, St. Petersburg State University, St. Petersburg, Russia; 4 Algorithmic Biology Lab, St. Petersburg Academic University, St.Petersburg, Russia; 5 Biological Data Management and Technology Center, Lawrence Berkeley National Laboratory, Berkeley, California, USA; 6 Oak Ridge National Laboratory, Oak Ridge, Tennessee, USA; 7 HZI – Helmholtz Centre for Infection Research, Braunschweig, Germany; 8 Leibniz Institute DSMZ - German Collection of Microorganisms and Cell Cultures, Braunschweig, Germany; 9 University of California Davis Genome Center, Davis, California, USA; 10 Australian Centre for Ecogenomics, School of Chemistry and Molecular Biosciences, The University of Queensland, Brisbane, Australia

**Keywords:** strictly aerobic, motile, rod-shaped, acetogenic, mesophilic, ‘*Acetobacter aurantius’*, *Xanthomonadaceae*, GEBA

## Abstract

*Frateuria aurantia* (ex Kondô and Ameyama 1958) Swings *et al*. 1980 is a member of the bispecific genus *Frateuria* in the family *Xanthomonadaceae*, which is already heavily targeted for non-type strain genome sequencing. Strain Kondô 67^T^ was initially (1958) identified as a member of ‘*Acetobacter aurantius*’, a name that was not considered for the approved list. Kondô 67^T^ was therefore later designated as the type strain of the newly proposed acetogenic species *Frateuria aurantia*. The strain is of interest because of its triterpenoids (hopane family). *F. aurantia* Kondô 67^T^ is the first member of the genus *Frateura* whose genome sequence has been deciphered, and here we describe the features of this organism, together with the complete genome sequence and annotation. The 3,603,458-bp long chromosome with its 3,200 protein-coding and 88 RNA genes is a part of the *** G****enomic* ***E****ncyclopedia of* ***Bacteria**** and* ***Archaea***** project.

## Introduction

Strain Kondô 67^T^, also known as G-6^T^ and as IFO 3245^T^ (= DSM 6220 = ATCC 33424 = NBRC 3245) is the type strain of the species *Frateuria aurantia* [1], the type species in the bispecific genus *Frateuria* [1]. Kondô 67^T^ was originally isolated from *Lilium auratum* Lindl and classified as a member of ‘*Acetobacter aurantius*’ from which it was reclassified 22 years later as the type strain of the type species of *Frateuria* [1]. The genus was named after the Belgian microbiologist Joseph Frateur (1903-1974) [1]; the species epithet is derived from the Neo-Latin adjective *aurantia*, referring to the gold-yellow color of the strain on MYP agar [1]. Strain Kondô 67^T^ was characterized as ‘acetogenic’ [2] and as containing triterpenoids of the hopane family [3]. Here we present a summary classification and a set of features for *F. aurantia* Kondô 67^T^, together with the description of the genomic sequencing and annotation.

## Classification and features

A representative genomic 16S rRNA gene sequence of strain Kondô 67^T^ was compared using NCBI BLAST [4,5] under default settings (e.g., considering only the high-scoring segment pairs (HSPs) from the best 250 hits) with the most recent release of the Greengenes database [6] and the relative frequencies of taxa and keywords (reduced to their stem [7]) were determined, weighted by BLAST scores. The most frequently occurring genera were *Dyella* (34.3%), *Rhodanobacter* (24.0%), *Frateuria* (19.6%), *Luteibacter* (11.9%) and *'Luteibactor'* (3.7%) (105 hits in total). Regarding the eleven hits to sequences from members of the species, the average identity within HSPs was 99.6%, whereas the average coverage by HSPs was 100.0%. Among all other species, the one yielding the highest score was *Dyella ginsengisoli* (EF191354), which corresponded to an identity of 98.2% and an HSP coverage of 99.0%. (Note that the Greengenes database uses the INSDC (= EMBL/NCBI/DDBJ) annotation, which is not an authoritative source for nomenclature or classification.) The highest-scoring environmental sequence was HM556321 ('insect herbivore microbiome plant biomass-degrading capacity *Atta colombica* colony N11 fungus garden top clone TIBW663'), which showed an identity of 99.7% and an HSP coverage of 97.2%. The most frequently occurring keywords within the labels of all environmental samples which yielded hits were 'soil' (5.9%), 'sediment' (2.5%), 'microbi' (1.8%), 'enrich' (1.5%) and 'vent' (1.3%) (145 hits in total). The most frequently occurring keyword within the labels of those environmental samples which yielded hits of a higher score than the highest scoring species was 'atta, biomass-degrad, capac, colombica, coloni, fungu, garden, herbivor, insect, microbiom, plant, top' (8.3%) (6 hits in total), reflecting some of the known features of the strain’s origin.

**Figure 1:**
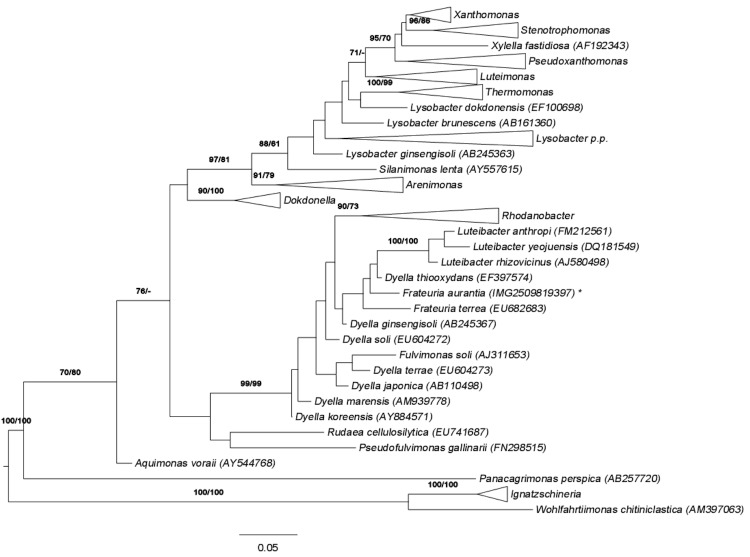
Phylogenetic tree highlighting the position of *F. aurantia* relative to the type strains of the other species within the family *Xanthomonadaceae*. The tree was inferred from 1,431 aligned characters [8,9] of the 16S rRNA gene sequence under the maximum likelihood (ML) criterion [10]. Rooting was done initially using the midpoint method [11] and then checked for its agreement with the current classification (Table 1). The branches are scaled in terms of the expected number of substitutions per site. Numbers adjacent to the branches are support values from 750 ML bootstrap replicates [12] (left) and from 1,000 maximum-parsimony bootstrap replicates [13] (right) if larger than 60%. Lineages with type strain genome sequencing projects registered in GOLD [14] are labeled with one asterisk, those also listed as 'Complete and Published' with two asterisks.

Figure 1 shows the phylogenetic neighborhood of *F. aurantia* in a 16S rRNA based tree. The sequences of the four identical 16S rRNA gene copies in the genome differ by one nucleotide from the previously published 16S rRNA sequence (AB091194).

**Table 1:** Classification and general features of *F. aurantia* Kondô 67^T^ according to the MIGS recommendations [15] (published by the Genome Standards Consortium [16]) and NamesforLife [17].

**MIGS ID**	**Property**	**Term**	**Evidence code**
	Current classification	Domain *Bacteria*	TAS [18]
		Phylum *Proteobacteria*	TAs [19]
		Class *Gammaproteobacteria*	TAS [20,21]
		Order *Xanthomonadales*	TAS [20,22]
		Family *Xanthomonadaceae*	TAS [20,22]
		Genus *Frateuria*	TAS [1,23]
		Species *Frateuria aurantia*	TAS [1]
		Type strain Kondô 67 = G-6 = IFO 3245	TAS [1]
	Gram stain	negative	TAS [1]
	Cell shape	rod-shaped, mostly strait	TAS [1]
	Motility	motile	TAS [1]
	Sporulation	not reported	
	Temperature range	mesophile	TAS [1]
	Optimum temperature	30°C	TAS [1]
	Salinity	0.2 - 2% NaCl (w/v)	TAS [1]
MIGS-22	Oxygen requirement	aerobe	TAS [1]
	Carbon source	glucose, yeast extract, mannitol, peptone	TAS [1]
	Energy metabolism	organoheterotroph	TAS [1]
MIGS-6	Habitat	*Lilium auratum*	TAS [1]
MIGS-15	Biotic relationship	host-associated	TAS [1]
MIGS-14	Pathogenicity	none	NAS
	Biosafety level	1	TAS [24]
MIGS-23.1	Isolation	from *Lilium auratum* Lindl	TAS [25]
MIGS-4	Geographic location	Kawasaki, Japan	TAS [1]
MIGS-5	Sample collection time	1958 or before	TAS [25]
MIGS-4.1	Latitude	35.50	TAS [1]
MIGS-4.2	Longitude	139.77	TAS [1]
MIGS-4.3	Depth	not reported	
MIGS-4.4	Altitude	not reported	

Evidence codes - TAS: Traceable Author Statement (i.e., a direct report exists in the literature); NAS: Non-traceable Author Statement (i.e., not directly observed for the living, isolated sample, but based on a generally accepted property for the species, or anecdotal evidence). Evidence codes are from the Gene Ontology project [26].

**Figure 2:**
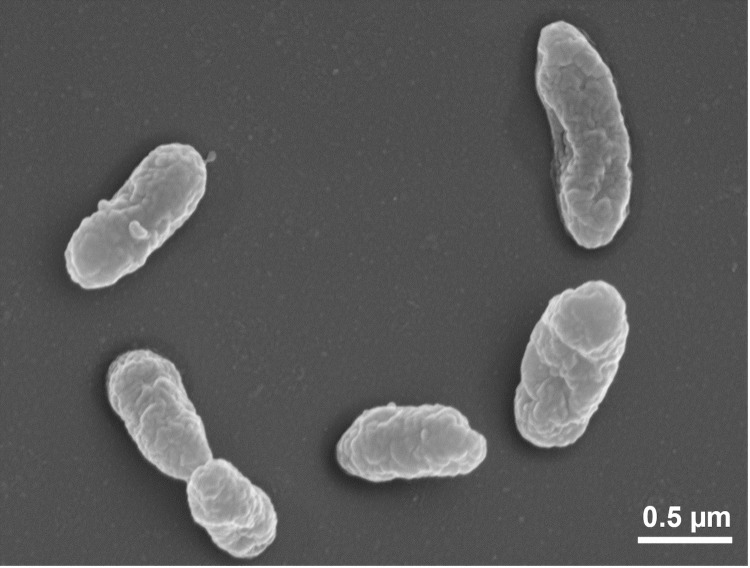
Scanning electron micrograph of *F. aurantia* Kondô 67^T^

*F. aurantia* Kondô 67^T^ cells stain Gram-negative [1], were straight rod shaped, 0.5-0.7 μm in width and 0.7-3.5 μm in length (Figure 2) [1] and motile via polar flagella [1] (not visible in Figure 2). Cells occur singly or in pairs, rarely in filaments [1]. Cultures grow in dark, glistening, flat colonies with a soluble brown pigment [1]. They are oxidase positive and catalase negative [1]; physiological features and antibiotic susceptibilities were reported in great detail in [1]. Cells grow well at pH 3.6 and 34°C [1].

### Chemotaxonomy

Besides trace amounts of diploptene and rearranged compounds like fern-7-ene [3], the main lipids isolated from DSM 6220^T^ are *iso*-branched fatty acids and triterpenoids of the hopane family, such as bacteriohopanetetrol and derived hopanoid. The organism also produces ubiquinone Q8 [27].

## Genome sequencing and annotation

### Genome project history

This organism was selected for sequencing on the basis of its phylogenetic position [28], and is part of the *** G****enomic* ***E****ncyclopedia of* ***Bacteria**** and* ***Archaea***** project [29]. The genome project is deposited in the Genomes On Line Database [14] and the complete genome sequence is deposited in GenBank. Sequencing, finishing and annotation were performed by the DOE Joint Genome Institute (JGI) using state of the art sequencing technology [30]. A summary of the project information is shown in Table 2.

**Table 2:** Genome sequencing project information

**MIGS ID**	**Property**	**Term**
MIGS-31	Finishing quality	Finished
MIGS-28	Libraries used	Two genomic libraries: one 454 PE library (7.5 kb insert size), one Illumina library
MIGS-29	Sequencing platforms	Illumina GAii, 454 GS FLX Titanium
MIGS-31.2	Sequencing coverage	537.4 × Illumina; 8.6 × pyrosequence
MIGS-30	Assemblers	Newbler version 2.3-PreRelease-6/30/2009, Velvet 1.0.13, phrap version SPS - 4.24
MIGS-32	Gene calling method	Prodigal
	INSDC ID	CP003350
	GenBank Date of Release	June 14, 2012
	GOLD ID	Gc02155
	NCBI project ID	64505
	Database: IMG	2509601034
MIGS-13	Source material identifier	DSM 6220
	Project relevance	Tree of Life, GEBA

### Growth conditions and DNA isolation

*F. aurantia* strain Kondô 67^T^, DSM 6220, was grown in DSMZ medium 360 (YPM medium) [31] at 30°C. DNA was isolated from 0.5-1 g of cell paste using standard procedures at the DSMZ DNA laboratory and quality control processes requested by the sequencing center (JGI). DNA is available through the DNA Bank Network [32].

### Genome sequencing and assembly

The genome was sequenced using a combination of Illumina and 454 sequencing platforms. All general aspects of library construction and sequencing can be found at the JGI website [33]. Pyrosequencing reads were assembled using the Newbler assembler (Roche). The initial Newbler assembly consisting of 36 contigs in one scaffold was converted into a phrap [34] assembly by making fake reads from the consensus, to collect the read pairs in the 454 paired end library. Illumina GAii sequencing data (2,074.3 Mb) was assembled with Velvet [35] and the consensus sequences were shredded into 1.5 kb overlapped fake reads and assembled together with the 454 data. The 454 draft assembly was based on 63.7Mb 454 draft data. Newbler parameters are -consed -a 50 -l 350 -g -m -ml 20. The Phred/Phrap/Consed software package [34] was used for sequence assembly and quality assessment in the subsequent finishing process. After the shotgun stage, reads were assembled with parallel phrap (High Performance Software, LLC). Possible mis-assemblies were corrected with gapResolution [33], Dupfinisher [36], or sequencing cloned bridging PCR fragments with subcloning. Gaps between contigs were closed by editing in Consed, by PCR and by Bubble PCR primer walks (J.-F. Chang, unpublished). A total of 43 additional reactions and one shatter library were necessary to close gaps and to raise the quality of the final sequence. Illumina reads were also used to correct potential base errors and increase consensus quality using a software Polisher developed at JGI [37]. The error rate of the final genome sequence is less than 1 in 100,000. Together, the combination of the Illumina and 454 sequencing platforms provided 546.0 × coverage of the genome. The final assembly contained 163,130 pyrosequence and 25,455,174 Illumina reads.

### Genome annotation

Genes were identified using Prodigal [38] as part of the DOE-JGI [39] genome annotation pipeline, followed by a round of manual curation using the JGI GenePRIMP pipeline [40]. The predicted CDSs were translated and used to search the National Center for Biotechnology Information (NCBI) non-redundant database, UniProt, TIGRFam, Pfam, PRIAM, KEGG, COG, and InterPro databases. These data sources were combined to assert a product description for each predicted protein. Additional gene prediction analysis and functional annotation were performed within the Integrated Microbial Genomes - Expert Review (IMG-ER) platform [41].

## Genome properties

**Figure 3:**
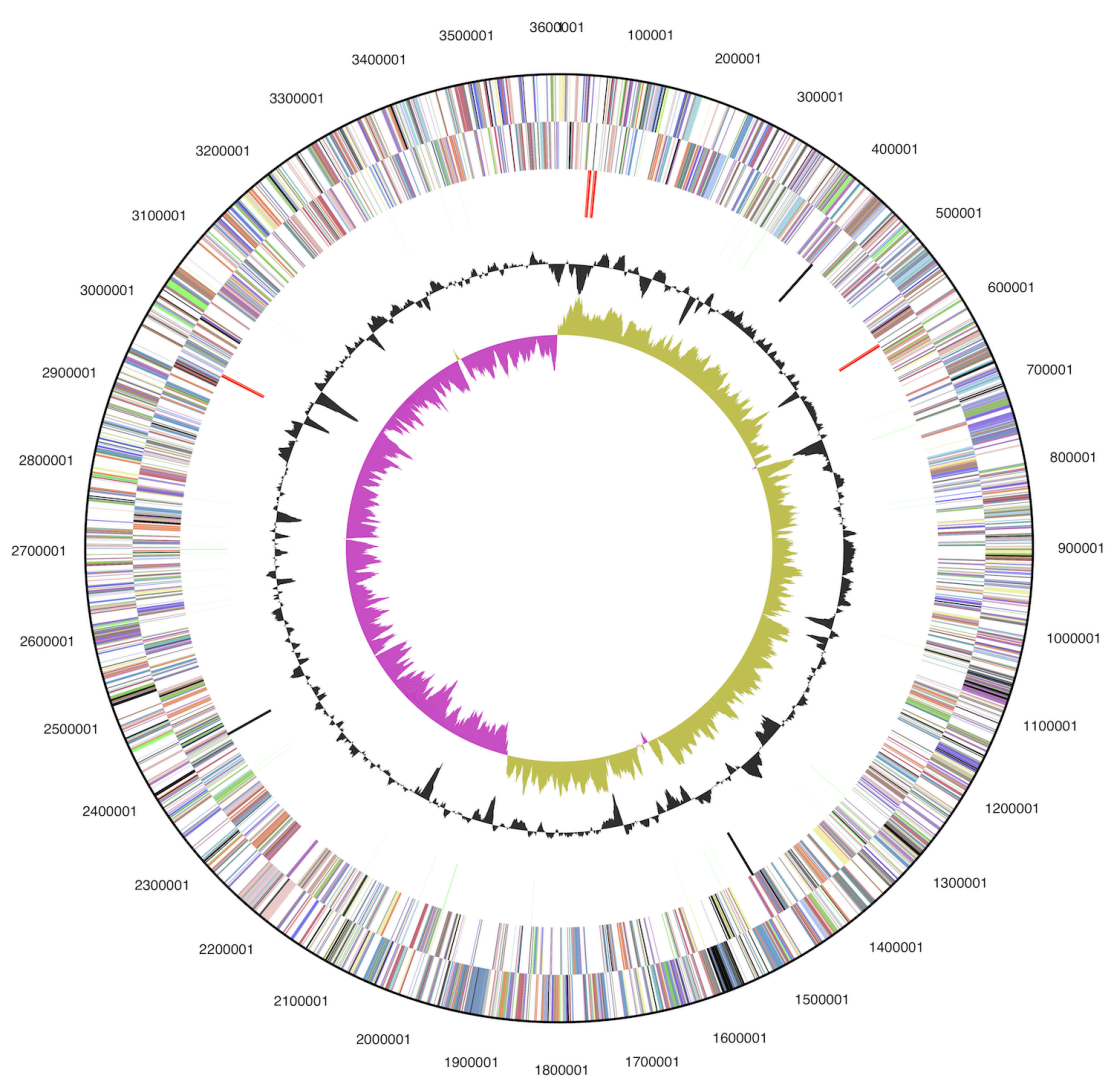
Graphical map of the chromosome. From outside to center: Genes on forward strand (colored by COG categories), Genes on reverse strand (colored by COG categories), RNA genes (tRNAs green, rRNAs red, other RNAs black), GC content(black), GC skew (purple/olive).

The genome consists of a 3,603,458 bp long circular chromosome with a G+C content of 63.4% (Table 3 and Figure 3). Of the 3,288 genes predicted, 3,200 were protein-coding genes, and 88 RNAs; 99 pseudogenes were also identified. The majority of the protein-coding genes (79.6%) were assigned a putative function while the remaining ones were annotated as hypothetical proteins. The distribution of genes into COGs functional categories is presented in Table 4.

**Table 3:** Genome Statistics

**Attribute**	Value	% of Total
Genome size (bp)	3,603,458	100.00%
DNA coding region (bp)	3,189,580	88.51%
DNA G+C content (bp)	2,284,441	63.40%
Number of replicons	1	
Extrachromosomal elements	0	
Total genes	3,288	100.00%
RNA genes	88	2.68%
rRNA operons	4	
tRNA genes	73	2.22%
Protein-coding genes	3,200	97.32%
Pseudo genes	99	3.01%
Genes with function prediction (proteins)	2,616	79.56%
Genes in paralog clusters	1,350	41.06%
Genes assigned to COGs	2,610	79.38%
Genes assigned Pfam domains	2,724	82.85%
Genes with signal peptides	313	9.52%
Genes with transmembrane helices	722	21.96%
CRISPR repeats	1	

**Table 4:** Number of genes associated with the general COG functional categories

**Code**	**value**	**%age**	**Description**
J	167	5.7	Translation, ribosomal structure and biogenesis
A	1	0.0	RNA processing and modification
K	192	6.6	Transcription
L	145	5.0	Replication, recombination and repair
B	1	0.0	Chromatin structure and dynamics
D	30	1.0	Cell cycle control, cell division, chromosome partitioning
Y	0	0.0	Nuclear structure
V	56	1.9	Defense mechanisms
T	129	4.4	Signal transduction mechanisms
M	214	7.3	Cell wall/membrane biogenesis
N	92	3.1	Cell motility
Z	0	0.0	Cytoskeleton
W	0	0.0	Extracellular structures
U	112	3.8	Intracellular trafficking and secretion, and vesicular transport
O	133	4.5	Posttranslational modification, protein turnover, chaperones
C	186	6.4	Energy production and conversion
G	170	5.8	Carbohydrate transport and metabolism
E	209	7.1	Amino acid transport and metabolism
F	68	2.3	Nucleotide transport and metabolism
H	143	4.9	Coenzyme transport and metabolism
I	101	3.5	Lipid transport and metabolism
P	146	5.0	Inorganic ion transport and metabolism
Q	63	2.2	Secondary metabolites biosynthesis, transport and catabolism
R	323	11.0	General function prediction only
S	246	8.4	Function unknown
-	678	20.6	Not in COGs

## References

[1] Swings J Gillis M Kersters K De Vos P Gosselé F de Ley J *Frateuria*, a new genus for "*Acetobacter aurantius*". Int J Syst Bacteriol 1980; 30:547-556 10.1099/00207713-30-3-547

[2] Johnson DB Rolfe S Hallberg KB Iversen E Isolation and phylogenetic characterization of acidophilic microorganisms indigenous to acidic drainage waters at an abandoned Norwegian copper mine. Environ Microbiol 2001; 3:630-637 10.1046/j.1462-2920.2001.00234.x 11722543

[3] Joyeux C Fouchard S Llopiz P Neunlist S Influence of the temperature and the growth phase on the hopanoids and fatty acids content of *Frateuria aurantia* (DSMZ 6220). FEMS Microbiol Ecol 2004; 47:371-379 10.1016/S0168-6496(03)00302-7 19712325

[4] Altschul SF Gish W Miller W Myers EW Lipman DJ Basic local alignment search tool. J Mol Biol 1990; 215:403-410 2231712 10.1016/S0022-2836(05)80360-2

[5] Korf I, Yandell M, Bedell J. BLAST, O'Reilly, Sebastopol, 2003.

[6] DeSantis TZ Hugenholtz P Larsen N Rojas M Brodie EL Keller K Huber T Dalevi D Hu P Andersen GL Greengenes, a chimera-checked 16S rRNA gene database and workbench compatible with ARB. Appl Environ Microbiol 2006; 72:5069-5072 10.1128/AEM.03006-05 16820507 PMC1489311

[7] Porter MF. An algorithm for suffix stripping. Program: *electronic library and information systems* 1980; **14**:130-137.

[8] Lee C Grasso C Sharlow MF Multiple sequence alignment using partial order graphs. Bioinformatics 2002; 18:452-464 10.1093/bioinformatics/18.3.452 11934745

[9] Castresana J Selection of conserved blocks from multiple alignments for their use in phylogenetic analysis. Mol Biol Evol 2000; 17:540-552 10.1093/oxfordjournals.molbev.a026334 10742046

[10] Stamatakis A Hoover P Rougemont J A rapid bootstrap algorithm for the RAxML web servers. Syst Biol 2008; 57:758-771 10.1080/10635150802429642 18853362

[11] Hess PN De Moraes Russo CA An empirical test of the midpoint rooting method. Biol J Linn Soc Lond 2007; 92:669-674 10.1111/j.1095-8312.2007.00864.x PMC7110036 32287391

[12] Pattengale ND Alipour M Bininda-Emonds ORP Moret BME Stamatakis A How many bootstrap replicates are necessary? Lect Notes Comput Sci 2009; 5541:184-200 10.1007/978-3-642-02008-7_13

[13] Swofford DL. PAUP*: Phylogenetic Analysis Using Parsimony (*and Other Methods), Version 4.0 b10. Sinauer Associates, Sunderland, 2002.

[14] Pagani I Liolios K Jansson J Chen IM Smirnova T Nosrat B Markowitz VM Kyrpides NC The Genomes OnLine Database (GOLD) v.4: status of genomic and metagenomic projects and their associated metadata. Nucleic Acids Res 2012; 40:D571-D579 10.1093/nar/gkr1100 22135293 PMC3245063

[15] Field D Garrity G Gray T Morrison N Selengut J Sterk P Tatusova T Thomson N Allen MJ Angiuoli SV The minimum information about a genome sequence (MIGS) specification. Nat Biotechnol 2008; 26:541-547 10.1038/nbt1360 18464787 PMC2409278

[16] Field D Amaral-Zettler L Cochrane G Cole JR Dawyndt P Garrity GM Gilbert J Glöckner FO Hirschman L Karsch-Mzrachi I PLoS Biol •••; 9:e1001088 10.1371/journal.pbio.1001088 21713030 PMC3119656

[17] Garrity G NamesforLife. BrowserTool takes expertise out of the database and puts it right in the browser. Microbiol Today 2010; 37:9

[18] Woese CR Kandler O Wheelis ML Towards a natural system of organisms. Proposal for the domains *Archaea* and *Bacteria.* Proc Natl Acad Sci USA 1990; 87:4576-4579 10.1073/pnas.87.12.4576 2112744 PMC54159

[19] Garrity GM, Bell JA, Lilburn T. Phylum XIV. *Proteobacteria* phyl. nov. *In:* Garrity GM, Brenner DJ, Krieg NR, Staley JT (*eds*), Bergey's Manual of Systematic Bacteriology, Second Edition, Volume 2, Part B, Springer, New York, 2005, p. 1.

[20] Validation of publication of new names and new combinations previously effectively published outside the IJSEM. List no. 106. Int J Syst Evol Microbiol 2005; 55:2235-2238 10.1099/ijs.0.64108-0

[21] Garrity GM, Bell JA, Lilburn T. Class III. *Gammaproteobacteria* class. nov. *In:* Garrity GM, Brenner DJ, Krieg NR, Staley JT (*eds*), Bergey's Manual of Systematic Bacteriology, Second Edition, Volume 2, Part B, Springer, New York, 2005, p. 1.

[22] Saddler GS, Bradbury JF. Order III. *Xanthomonadales* ord. nov. *In:* Garrity GM, Brenner DJ, Krieg NR, Staley JT (*eds*), Bergey's Manual of Systematic Bacteriology, Second Edition, Volume 2, Part B, Springer, New York, 2005, p. 63.

[23] Zhang JY Liu XY Liu SJ *Frateuria terrea* sp. nov., isolated from forest soil, and emended description of the genus *Frateuria.* Int J Syst Evol Microbiol 2011; 61:443-447 10.1099/ijs.0.021618-0 20348318

[24] BAuA. 2010, Classification of bacteria and archaea in risk groups. http://www.baua.de TRBA 466, p. 89.

[25] Kondô K Ameyama M Carbohydrate metabolism by *Acetobacter* species. I. Oxidative activity for various carbohydrates. Bull Agric Chem Soc Jpn 1958; 22:369-372 10.1271/bbb1924.22.369

[26] Ashburner M Ball CA Blake JA Botstein D Butler H Cherry JM Davis AP Dolinski K Dwight SS Eppig JT Gene ontology: tool for the unification of biology. The Gene Ontology Consortium. Nat Genet 2000; 25:25-29 10.1038/75556 10802651 PMC3037419

[27] Yamada Y Okada Y Kondô K Isolation and characterization of “polarly flaggelated intermediate strains” in acetic bacteria. J Gen Appl Microbiol 1976; 22:237-245 10.2323/jgam.22.237

[28] Klenk HP Göker M *En route* to a genome-based classification of *Archaea* and *Bacteria?* Syst Appl Microbiol 2010; 33:175-182 10.1016/j.syapm.2010.03.003 20409658

[29] Wu D Hugenholtz P Mavromatis K Pukall R Dalin E Ivanova NN Kunin V Goodwin L Wu M Tindall BJ A phylogeny-driven Genomic Encyclopedia of *Bacteria* and *Archaea.* Nature 2009; 462:1056-1060 10.1038/nature08656 20033048 PMC3073058

[30] Mavromatis K Land ML Brettin TS Quest DJ Copeland A Clum A Goodwin L Woyke T Lapidus A Klenk HP The fast changing landscape of sequencing technologies and their impact on microbial genome assemblies and annotation. PLoS ONE 2012; 7:e48837 10.1371/journal.pone.0048837 23251337 PMC3520994

[31] List of growth media used at DSMZ: http://www.dsmz.de/catalogues/catalogue-microorganisms/culture-technology/list-of-media-for-microorganisms.html

[32] Gemeinholzer B Dröge G Zetzsche H Haszprunar G Klenk HP Güntsch A Berendsohn WG Wägele JW The DNA Bank Network: the start from a German initiative. Biopreserv Biobank 2011; 9:51-55 10.1089/bio.2010.0029 24850206

[33] The DOE Joint Genome Institute www.jgi.doe.gov

[34] Phrap and Phred for Windows. MacOS, Linux, and Unix. www.phrap.com

[35] Zerbino DR Birney E Velvet: algorithms for de novo short read assembly using de Bruijn graphs. Genome Res 2008; 18:821-829 10.1101/gr.074492.107 18349386 PMC2336801

[36] Han C, Chain P. Finishing repeat regions automatically with Dupfinisher. *In:* Proceedings of the 2006 international conference on bioinformatics & computational biology. Arabnia HR, Valafar H (*eds*), CSREA Press. June 26-29, 2006: 141-146.

[37] Lapidus A, LaButti K, Foster B, Lowry S, Trong S, Goltsman E. POLISHER: An effective tool for using ultra short reads in microbial genome assembly and finishing. AGBT, Marco Island, FL, 2008.

[38] Hyatt D Chen GL Locascio PF Land ML Larimer FW Hauser LJ Prodigal Prokaryotic Dynamic Programming Genefinding Algorithm. BMC Bioinformatics 2010; 11:119 10.1186/1471-2105-11-119 20211023 PMC2848648

[39] Mavromatis K Ivanova NN Chen IM Szeto E Markowitz VM Kyrpides NC The DOE-JGI Standard operating procedure for the annotations of microbial genomes. Stand Genomic Sci 2009; 1:63-67 10.4056/sigs.632 21304638 PMC3035208

[40] Pati A Ivanova N Mikhailova N Ovchinikova G Hooper SD Lykidis A Kyrpides NC GenePRIMP: A Gene Prediction Improvement Pipeline for microbial genomes. Nat Methods 2010; 7:455-457 10.1038/nmeth.1457 20436475

[41] Markowitz VM Ivanova NN Chen IMA Chu K Kyrpides NC IMG ER: a system for microbial genome annotation expert review and curation. Bioinformatics 2009; 25:2271-2278 10.1093/bioinformatics/btp393 19561336

